# Assessment Method for a Power Analysis to Identify Differentially Expressed Pathways

**DOI:** 10.1371/journal.pone.0037510

**Published:** 2012-05-18

**Authors:** Shailesh Tripathi, Frank Emmert-Streib

**Affiliations:** Computational Biology and Machine Learning Lab, Center for Cancer Research and Cell Biology, School of Medicine, Dentistry and Biomedical Sciences, Queen's University Belfast, Belfast, United Kingdom; National Institutes of Health, United States of America

## Abstract

Gene expression data can provide a very rich source of information for elucidating the biological function on the pathway level if the experimental design considers the needs of the statistical analysis methods. The purpose of this paper is to provide a comparative analysis of statistical methods for detecting the differentially expression of pathways (DEP). In contrast to many other studies conducted so far, we use three novel simulation types, producing a more realistic correlation structure than previous simulation methods. This includes also the generation of surrogate data from two large-scale microarray experiments from prostate cancer and ALL. As a result from our comprehensive analysis of 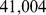 parameter configurations, we find that each method should only be applied if certain conditions of the data from a pathway are met. Further, we provide method-specific estimates for the optimal sample size for microarray experiments aiming to identify DEP in order to avoid an underpowered design. Our study highlights the sensitivity of the studied methods on the parameters of the system.

## Introduction

The functional analysis of high-throughput data is a challenging but promising direction in the post-genomics era. It is challenging because genome-wide data are high-dimensional and noisy, but promising due to its potential to reveal knowledge about the systemic working mechanisms of biological information processing within cells, which we are currently lacking [1]–[4].

In the context of expression data the interest shifted in recent years from approaches focusing on the analysis of individual genes, detecting their differentially expression [5]–[7], toward the analysis of gene sets in order to identify differentially expressed sets of genes [8]–[11]. The rational behind this is that genes and their products do not work in isolation but interact with each other in a concerted manner in order for a phenotype to emerge [12]. Many univariate, multivariate and nonparametric statistical methods have been either newly developed or existing methodological techniques have been adapted for this problem [13]–[16]. One important property that allows to distinguish different types of such hypotheses tests was discussed in [17]. There, tests have been distinguished based on the data used for the comparison. A hypothesis test comparing a gene set to all other gene sets available is called *competitive*, whereas a test comparing the same gene set for two different phenotypes is called *self-contained*.

The name ‘*gene set*’ associated with the above methods implies that the choice for defining gene sets by populating them with specific genes is not constraint. However, in the present study, we are considering only gene sets that have been defined by using biological information regarding their association with specific pathways extracted, e.g., from the gene ontology database [18] or KEGG [19]. For this reason we refer to them in the following as *pathway-based methods* [20] to indicate this explicitly.

The major goal of this paper is to compare two self-contained (*sum of t-square* and Hotelling's 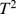 [21], [22]) and one competitive test (GSEA [23]) with each other, for a variety of simulated and biological expression data, in order to gain insights into the dependence of the power of these methods on the correlation structure among genes. The reason for selecting these tests is their complementary nature, representing univariate (*sum of t-square*), multivariate (Hotelling's 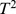) as well as competitive and self-contained tests. In [24] it has been shown that there are currently only about three different null hypotheses effectively tested among all self-contained tests, which include the null hypothesis of the *sum of t-square* test and Hotelling's 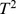. Because GSEA is a competitive test, its null hypothesis is conceptually different to the above ones making the three selected methods complementary to each other with respect to the tested null hypotheses. The reason for choosing GSEA over other competitive methods, which are based on methodological extensions [25], [26], is the popularity of this method especially among biologists [27], and the vast number of studies it has been already used for.

In contrast to the many studies that have been conducted so far investigating the power of methods for identifying differentially expressed pathways, we are focusing on the *correlation structure* in gene expression data. Other studies conducted in this context either did not consider the correlation among genes [25], [28], assumed a constant [13], [29], a random [30], an autoregressive [31] or a compound symmetry correlation structure among genes [14], [32], [33] or studied real microarray data only [34]–[36], which do not allow to study different configurations by adjusting model parameters. The main problem with the above simulation studies is that they do not provide a realistic correlation structure among genes. The reason for this is that the made assumptions do not lead to a gene network-like correlation structure as observed for gene expression data. Technically, this means that the inverse of the covariance matrix does not reflect the independence relations that can be found in such network structures, as we will discuss in detail in section ‘Simulation of network-like correlation structure’. However, due to the fact that we are only considering gene sets that correspond to biological pathways, the strength of the correlation and its structure are important parameters that need to be controlled properly in order to make the transition from gene sets to pathways. In order to overcome this severe limitation, two algorithms have been developed, in a different context, for generating a covariance matrix for a multivariate normal distribution whose inverse is consistent with the independence relations of a network [37], [38]. In order to generate such a covariance matrix, both methods need a network as an input for their algorithm. For our simulation study, we employ both algorithms for generating simulated expression data with a gene network-like correlation structure by using a protein interaction network and a transcriptional regulatory network from yeast as input network. This provides a biologically realistic constraint on the resulting correlation structure. In the following we call these simulation types III and IV. Here by ‘*biologically realistic*’ we mean that an experimentally determined protein interaction network and a transcriptional regulatory network are more realistic than artificially generated network structures using a statistical method. In order to conduct a comprehensive analysis of important system parameters, we include in our study also the influence of the sample size, detection call, i.e., the percentage of genes that is differentially expressed within a pathway, and the pathway size on the identification of differentially expressed pathways.

In addition to simulated expression data, we use also two large-scale cancer data sets from DNA microarray experiments [39], [40]. By applying a bootstrap approach [41], [42], we use these data sets to generate surrogate data of smaller sample sizes. This allows us to study the robustness of the statistical methods over a wide range of realistic sample sizes without the need for making assumptions about the underlying pathology of the pathways in order to declare, e.g., pathways as true positives. Further, we compare the correlation structure of simulated and biological pathways as defined via the gene ontology database [18].

## Methods

### Pathway-based method

#### GSEA

This method was introduced by [11], [23] in order to identify the differential expression of predefined gene sets. GSEA is considered a competitive test [17] because it compares a test set to a background data set. Let 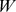 be the set of genes to be tested and 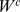 its complement in a way that the union of both sets gives all genes, i.e., 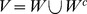. Briefly, GSEA consists of the following steps, applied to each pathway:

1. Estimation of gene-wise test statistics.
2. Rank ordering of the test statistics.
3. Calculation of an enrichment score (ES) for a pathway.
4. Permutation of the gene-labels to estimate the significance of the enrichment score (p-value) for the pathway.

The hypotheses tested by GSEA are:

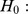
 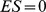 - vanishing test score

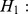
 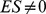 - non-vanishing test score

#### Hotelling's 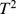


The Hotelling 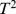 test is a self-contained test that is a multivariate generalization of the univariate t-test. Its null and alternative hypothesis can be formulated as:

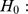
 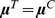 - equality of the p-dimensional population mean vectors

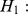
 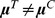 - difference of the p-dimensional population mean vectors

Suppose we have two groups with 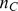 samples from the control group and 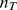 samples for the treatment group, each consisting of 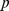 genes. Let the expression level of the 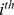 sample of the control group and treatment group be given by 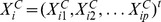 and 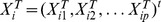, respectively. The pooled covariance matrix 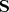 is then defined by
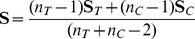
(1)where 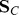 and 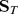 are the covariance matrices for the control and treatment group. Hotelling's 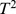 is defined as
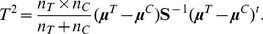
(2)The inverse of the covariance matrix is estimated via the shrinkage estimator [43]–[46]. The statistical significance of the test statistic 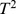 is estimated from sample-label permuted data.

#### Sum of t-square

The *sum of t-square* test is an univariate test based on t-scores, 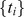, obtained for each of the 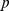 genes individually for a given set [22]. The test statistic for each pathway is given by
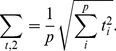
(3)Its null and alternative hypothesis can be formulated as:

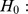
 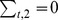 - vanishing test score

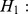
 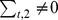 - non-vanishing test score

Again, the significance of 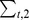 is assessed from sample-label permuted data.

### Simulation algorithms

In order to assess the performance of the statistical methods we use three principally different algorithms to simulate expression data.

#### Simulation of uncorrelated data

For this method we, first, define different non-overlapping pathways of varying sizes including a total of 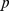 genes. Then we draw iid (Independent and identically distributed) samples from a standard normal distribution, i.e., 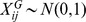, for each gene 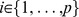 and sample 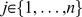, for the control (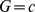) and treatment (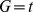) group. In order to make a difference between the control and treatment group we add a constant factor of one to a certain percent of genes of all pathways for the treatment group.

#### Simulation of correlated data

First, we generate a matrix 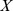 with 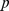 rows and 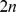 columns with a sample size of 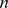 for the control and treatment group, i.e., 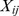 with 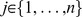 corresponds to the control and 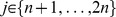 to the treatment group. Each component of 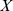 is independently sampled from a standard normal distribution, i.e., 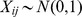. Then we generate a 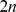-dimensional random vector 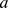 whose components are also iid drawn from the standard normal distribution. Define
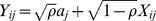
(4)where 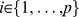 and 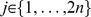, so that the average correlation between the genes (rows of 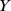) is 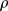 [29]. To model differential expressed pathways we add a constant factor of one to a certain percent of genes for the treatment group.

#### Simulation of a network-like correlation structure

For random variables that are from a p-dimensional multivariate normal distribution, i.e., 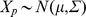, a simple relation between the components of the inverse covariance matrix 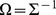 (also called precision or concentration matrix) and the conditional partial correlation holds [47]

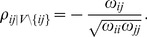
(5)Here 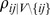 is the partial correlation coefficient between gene 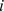 and 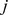 conditioned on all remaining genes and 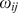 are the components of 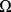. That means if 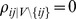 then gene 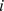 and 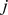 are independent from each other,
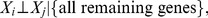
(6)if and only if 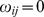. A multivariate normal distribution that is *Markov* with respect to an undirected network 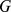 is called a *Gaussian graphical model*. This means that all conditional independence relations that can be found in 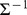 hold also in 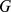 [47]. Hence, such a 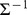 can be considered as consistent with all conditional independence relations in 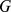.

We use the following two algorithms to obtain a covariance matrix for a given network structure 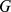.

1. The algorithm of [38] is based on successive orthogonal projections constraint by the network structure 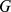, resulting in a consistent covariance matrix 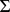.
2. The algorithm of [37], [48] is based on proportional iterative fitting [47] to enforce an average correlation in the data resulting in a covariance matrix 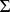 consistent with the conditional independence relations in 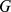.

As input network 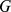 for these algorithms we use two yeast networks. The first is a protein-protein interaction network provided by the *Biogrid* database [49] and the second is a transcriptional regulatory network [50]. From both networks, we extract the giant connected component. The reason for selecting these networks is that a protein and a transcriptional regulatory network represent *observed* interaction structures among genes and gene product and, hence, provide a more realistic structure than artificially generated networks, e.g., by using the *preferential attachment* model to generate scale-free networks [51]. Once a covariance matrix 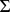 from one of the above algorithms is obtained, we use 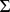 to generate iid samples from a multivariate normal distribution, i.e., 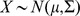. In order to simulate the differentially expression of pathways, we use a p-dimensional mean vector of zero, 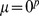, for the control group, and a mean vector 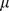 consisting of 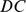 genes with an expression of 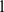 and 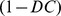 genes with an expression of 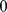 for the treatment group. For both groups, we use the same covariance matrix.

We would like to note that due to the properties of the Gaussian graphical model, as discussed above, this model has been used to infer gene regulatory networks from expression data [46], [52]–[54]. This indicates that the relation between the components of the inverse of the covariance matrix and the independence relations found in a network structure are generally considered to be biologically connected with each other for expression data. Hence, this provides a justification to consider the correlation structure generated from a Gaussian graphical model as biologically plausible.

### Simulation types

For our analysis we are using four different simulation types (ST) based on the algorithms described above. Because GSEA is a *competitive test* [17] it requires a background data set against which a pathway is compared. For ST I we simulate such a background data set explicitly. This background data consists of 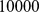 genes with expression values sampled from a normal distribution 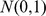, for both conditions, and a global correlation structure is imposed by Eqn. 4. For ST II–IV we use, instead, the remaining data *excluding* the pathway under investigation, as background data.

#### Simulation type I

For this type of simulation we generate simulated expressed data for all 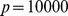 genes simultaneously, as described in methods section ‘Simulation of uncorrelated data’ and ‘Simulation of correlated data’, for which we define non-overlapping pathways of sizes ranging from 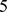 to 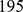 (step size 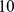), 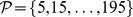. For each of these 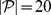 different pathway sizes we generate 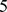 pathways, resulting in a total of 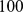 different non-overlapping pathways that contain in total 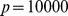 genes. Parameters studied: We study the influence of the sample size (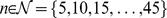, 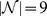), detection call (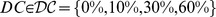, 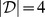) and of the correlation (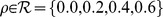, 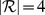). Here, the detection call (DC) [24] refers to the percent of differentially expressed genes in a pathway and 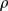 refers to the correlation between all genes in the overall set. This gives 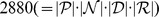 different parameter configurations. For GSEA, we generate a background data set *without expression difference* between the treatment and control group, i.e., 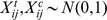.

#### Simulation type II

Here we generate simulated data separately for each pathway, as described in methods section ‘Simulation of correlated data’. In contrast to ST I, ST II generates a correlation among the genes within a pathway. We use an overall set of 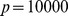 genes to define non-overlapping pathways, as for ST I. Parameters studied: We study the influence of the sample size (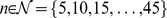, 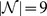), detection call (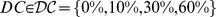, 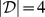) and of the correlation (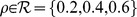, 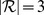). Here, 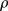 refers to the correlation for the genes within a pathway, whereas the average correlation among all genes is about zero. This gives 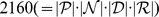 parameter configurations.

#### Simulation type III and IV

For this ST we generate simulated expressed data by sampling from a p-dimensional Gaussian graphical model. In order to obtain a more realistic correlation structure we use two different algorithms, as described in methods section ‘Simulation of network-like correlation structure’ in combination with a protein interaction network and a transcriptional regulatory network. We used the *gene ontology* database [18] to map the proteins to their corresponding *biological process* for level 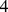. From this information we selected 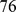 different but overlapping pathways that consist in total of 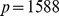 genes for algorithm (1) and 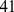 pathways for 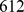 genes for algorithm (2). For the transcriptional regulatory network we select 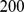 different but overlapping pathways that consist in total of 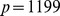 gene for algorithm (1) and (2). Parameters studied for both algorithms: We study the influence of the sample size (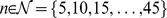, 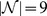) and the detection call (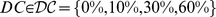, 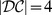). The overall average correlation between all genes is approximately zero. In addition, for algorithm (2) we study also different values of the correlation, (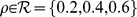, 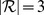). This gives 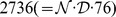 (ST III) and 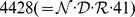 (ST IV) different parameter configurations for the protein interaction network and 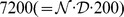 (ST III) and 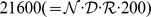 (ST IV) different parameter configurations for the transcription regulatory network.

We would like to note that in the results section, the estimates for the *false positive rate* (FPR) have been obtained by setting 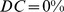, which corresponds to the case of no differentially expressed pathways [15], [24], [25].

### Surrogate data: ALL and prostate cancer

To assess the power of the three pathway-based methods for microarray data we use two different large-scale data sets based on Affymetrix chips. The first is a prostate cancer data set consisting of 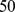 control samples and 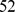 tumor samples [40]. The second data set is from B-cells derived from Acute lymphoblastic leukemia (ALL) [39]. From the entire data set we select 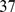 samples from the BCR/ABL group and 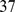 from the NEG group. For the preprocessing and normalization of these data sets we followed [39], [40]. After the normalization, we map the genes to the category *biological process* of level four in the gene ontology database [18] in order to obtain information about their association to biological pathways. For prostate cancer we obtain 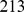 different pathways and for ALL 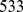.

Our analysis of these data consists of two steps. In the first step, we generate a reference list by testing the significance of pathways for the total number of samples 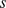 (prostate: 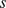 = 102, ALL: 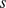 = 74). For the following analysis we use the results from this analysis as reference, because we consider the significant pathways as *true positives* and the nonsignificant pathways as *true negatives*. In the second step, we construct 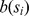 bootstrap data sets for various sample sizes, 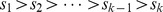, each data set drawn from the total of 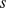 available samples. For each bootstrap data set, each method is applied and a p-value obtained for each pathway. From this, a result is assessed as true positive, true negative, false positive or false negative with respect to the reference list obtained for sample size 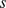 (step one). Due to the fact that our reference list may contain false declarations, our results assess the statistical robustness of the methods providing estimates for, e.g., their power, rather than their true value. Further, because we generate bootstrap data sets for each sample size 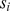, we consider these as surrogate data for newly generated data from independent experiments, which are not available.

## Results

For the following simulations, we use a significance level of 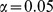.

### Simulation type I and II

**Figure 1:**
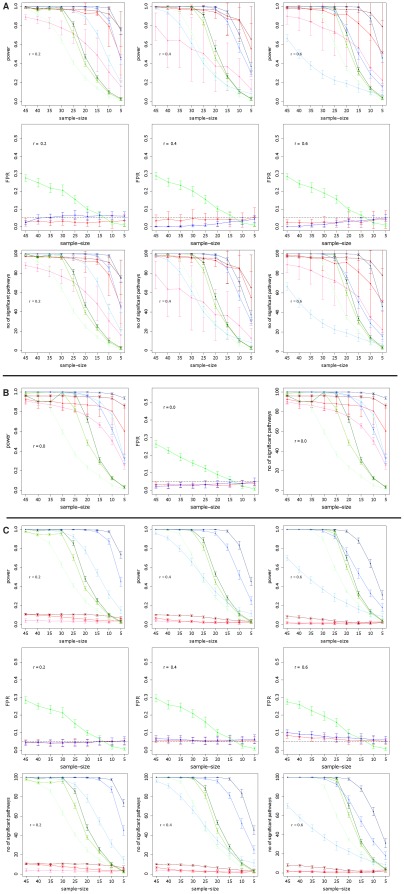
Simulation type I (A, B) and II (C): Power, FPR and number of significant pathways for GSEA (red), *sum of t-square* (blue) and Hotelling's 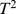 (green). DC = 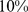 (light color), 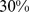 (medium color), 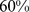 (dark color).

In Fig. 1 A-C we show the power for GSEA (red curves), Hotelling's 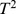 (green curves) and *sum of t-square* (blue curves) for simulation type I and II and different parameter settings. Here by the power we mean the probability that the statistical hypothesis test is rejected when the null hypothesis is truly false [55]. Practically, we estimate this probability by the population mean over repeated simulations [24]. The different color shadings code for different DC values; DC = 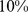 (light color), 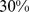 (medium color), 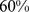 (dark color). In these figures, a ‘dot’ corresponds to a mean value, and the error bars refer to its standard deviation obtained from 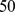 bootstrap samples. Each figure is indexed by the strength of the mean correlation.

For ST I (Fig. 1 A) the correlation has a much stronger influence on the power of *sum of t-square* and GSEA than on Hotelling's 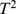, although Hotelling's 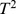 has generally a lower power. Also, the influence of the detection call is for the *sum of t-square* and GSEA strongest resulting in a considerable loss in power for 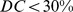. Hotelling's 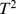 appears to be relative insensitive against different values of 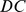. For high correlations and 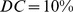 the *sum of t-square* test has by far the worst power. For ST II (Fig. 1 C) GSEA performs significantly worse for all values of DC, compared to simulation type I, showing an almost complete break down. The power of *sum of t-square* and Hotelling's 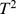 are comparable to the results for ST I. Regarding the number of significant pathways detected by the three methods, one can see that the *sum of t-square* test declares consistently more pathways as significant than any other method, for all conditions, except for very small sample sizes (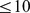) for ST I. In this case GSEA declares more pathways as significant. In general, the lower the DC value the lower the number of pathways declared as significant, whereas lower values having a stronger influence. Further, it is interesting to note that for 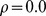 (Fig. 1 B) the *sum of t-square* and Hotelling's 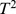 are different from each other despite that fact that in this case both tests should provide similar result, because the pooled covariance matrix 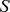 (see Eqn. 1) becomes diagonal. This indicates a poor behavior of the shrinkage estimator.

For ST II GSEA declares considerably less pathways as significant compared to the other methods, for all conditions. Regarding the false positive rate (FPR), GSEA and *sum of t-square* show a good control of the FPR at a significance level of 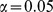. This is in contrast to Hotelling's 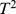 which has even for large sample sizes a FPR larger than 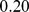. In order to find the cause for this behavior we split the pathways into two categories. In the first category we put all pathways having less than 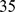 genes, in the second category we place all larger pathways (results not shown). From this analysis we find that also Hotelling's 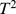 controls the FPR, but only for pathway sizes less than 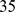. The reason for this behavior is related to the estimation of the inverse of the covariance matrix, 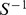, on which Hotelling's 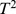 is based. For smaller pathways, their number of genes, 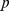, is closer to the number of samples, 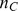 and 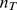, and, hence, the estimates for 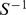 are more accurate than for larger pathways. Hence, for larger pathways one would need to improve the shrinkage estimator. Figure 1 B shows the result for uncorrelated data (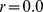). In this case ST I and II coincide with each other. In general, for the uncorrelated case the power is slightly higher for all methods and also the number of pathways declared significant increases.

### Simulation type III and IV

**Figure 2:**
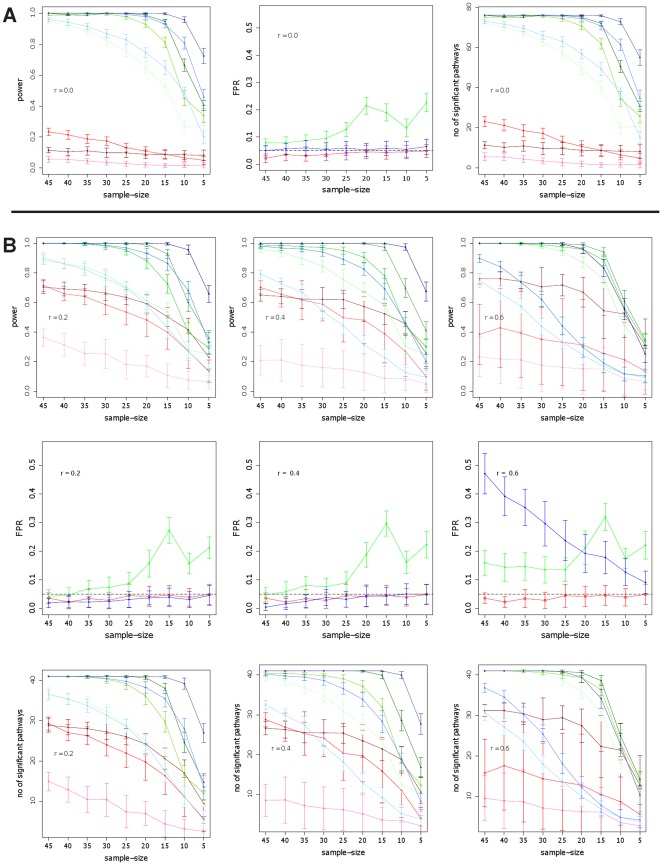
Simulation type III (A) and IV (B): Power, FPR and number of significant pathways for GSEA (red), *sum of t-square* (blue) and Hotelling's 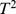 (green). DC = 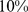 (light color), 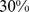 (medium color), 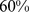 (dark color). Simulated data are from the protein network of yeast [49].

Fig. 2 (A and B) shows the results for simulation type III and IV. Here the *sum of t-square* and Hotelling's 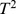 perform much better than GSEA. Interestingly, for ST IV and high correlations (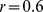) and small sample sizes (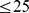) the power of Hotelling's 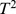 is even slightly higher than for the *sum of t-square* test and, more importantly, it is more robust with respect to DC values less than 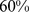. For the number of significant pathways we find again that GSEA declares less pathways as significant. Hotelling's 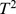 does not control well the false positive rate for small sample sizes (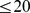) and has more problems in controlling the FPR with high correlations. For the *sum of t-square* the FPR is in general controlled, except for ST IV and 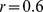. It is of interest to note that GSEA is the only method that controls the FPR for all conditions well.

The significant reduction of the power for GSEA for simulation type I compared to simulation type II to IV can be explained by the background data that have been generated for simulation type I, but not for the other simulation types. Due to the fact that GSEA is a competitive hypothesis test which estimates a test statistics w.r.t the background, the background data have a prominent influence on the power of GSEA whereas a large background dataset without expression changes in the conditions increases the sensitivity of this test.

**Figure 3:**
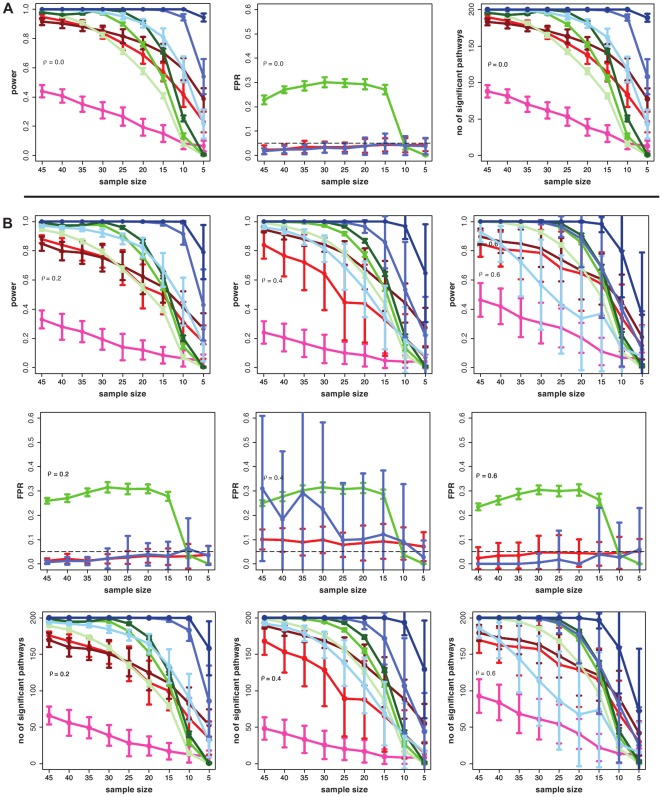
Simulation type III (A) and IV (B) : Power, FPR and *number of significant pathways* for GSEA (red), *sum of t-square* (blue) and Hotelling's 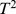 test (green). DC = 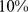 (light color), 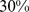 (medium color), 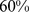 (dark color). Simulated data are from the transcriptional regulatory network of yeast [50].

In Fig. 3, we show similar results as in Fig. 2, however, for the transcriptional regulatory network of yeast [50] instead of the protein network, to generate simulated data. Overall, these results have a large resemblance to the results in Fig. 2. This demonstrates that structural properties of both networks on the pathway level are sufficiently similar to each other to result in similar results for the pathway methods. This corresponds, e.g., to the known similarity of the scale-free degree distribution of these networks [56].

### Surrogate data: ALL and prostate cancer

**Figure 4:**
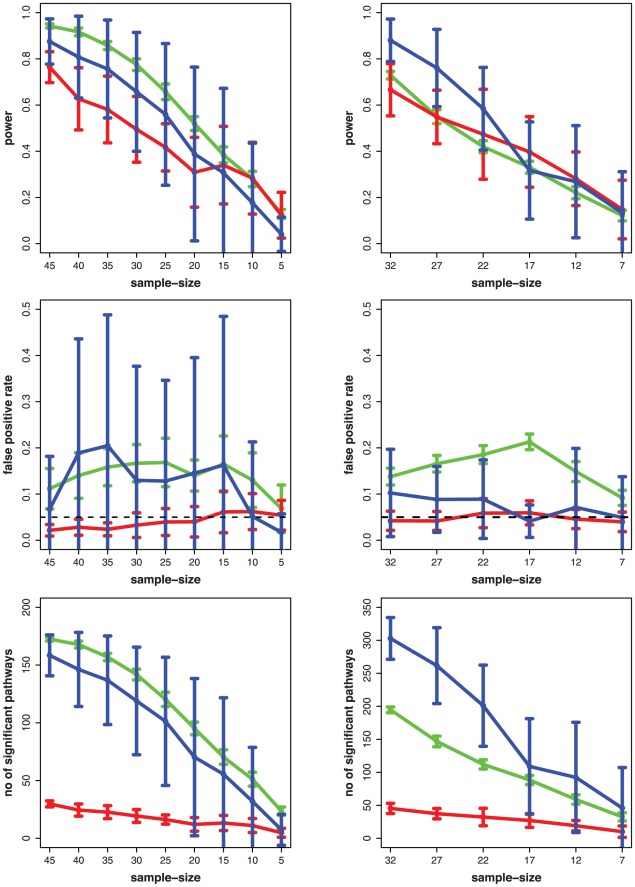
Left column: prostate cancer. Right column: ALL. Power, false positive rate and number of significant pathways for GSEA (red), *sum of t-square* (blue) and Hotelling's 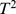 (green).

The results for prostate cancer (left) and ALL (right) are shown in Fig. 4. We want to re-emphasize that we used the pathways declared as significant for the total number of samples (prostate: 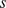 = 102, ALL: 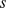 = 74) as a reference list. Hence, the power is related to these pathways and not to the *truely* expressed pathways. Similarly, the interpretation for the FPR and the number of significant pathways. On the first sight, all methods seem to perform similarly, although, GSEA has for high sample sizes the lowest power. However, Hotelling's 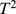 and *sum of t-square* declare for all studied sample sizes many more pathways as significant than GSEA. Similar to the simulation studies, GSEA controls the FPR well whereas the other two methods assume larger values.

An interesting observation of the power is its rapid decay for an even slightly reduced sample size. More precisely, for prostate cancer the total sample size is 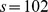 for which we identified the set of significant pathways we consider as ‘true positives’. However, when using only 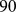 bootstrap samples (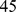 samples per condition) then the power of all three methods is already smaller than 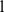, see Fig. 4. Even more severe effects are observed for ALL, where the total sample size is 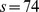, and results for 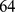 bootstrap samples (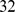 samples per condition) show a clearly reduced power. This lack of robustness for the initial sample sizes (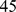 for prostate cancer and 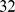 for ALL) suggests that the available *total* sample sizes are too small for the employed test statistics, because otherwise we would observe a stable plateau as in Fig. 1, 2 and 3, where a slight reduction of the sample size does not influence the power at all. Hence, from this decay, one can conclude that the total number of samples (prostate: 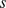 = 102, ALL: 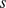 = 74) of both cancer data sets (ALL and prostate cancer) is not sufficiently large for the point estimator of the power to converge. This hints to a refinement of the experimental design of studies aiming to detect the DEP to avoid a study that is underpowered.

**Figure 5:**
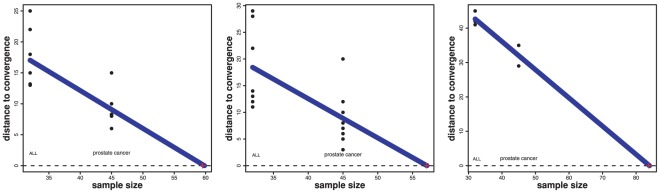
Left: Hotelling's 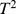, Middle: *sum of t-square*, Right: GSEA. The regression line is used to predict the *optimal* sample size (red cross) found from the intersection of the regression line with the horizontal dashed line corresponding to a ‘zero distance to convergence’.

In order to quantify this observation, we performed a linear regression analysis. For this analysis we use the size of the microarray experiments as predictor variable and the initial step size of the power curves (Fig. 4) as outcome variable, measured by its *distance to convergence*, as found from the comparison with our simulation results in Fig. 2 and 3. That means from Fig. 2 and 3 we obtain the minimal sample sizes for which the power reaches ‘1’, and the *initial step* size of the power corresponds to the power for 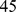 (prostate cancer) and 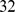 (ALL) samples from Fig. 4. We are only using the results from ST III and IV for this comparison, because they resemble more closely the correlation structure of real microarray data. We conduct a separate analysis to predict the optimal sample size for each method, see Fig. 5. Here *optimal* refers to the minimal sample size for a method to become invariant against the removal of a small number of samples.

For the regression, we obtain F-statistics (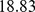, 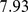 and 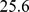) for the three linear regressions, in the order of the figures in 5, which are all significant with p-values of 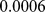, 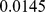 and 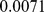. As a result of this analysis, we predict a sample size of 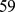 for Hotelling's 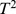 and 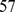 for the *sum of t-square* test (red crosses in Fig. 5). Due to the fact that for GSEA, its power does not converge in our simulation study for ST III and IV, we cannot make a prediction for this method. If we use the results form ST I instead, we obtain an estimated sample size of 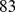 for GSEA. We would like to emphasize that we consider these estimates as optimistic and, hence, as lower bounds for optimal sample sizes since the simulations constitute only approximations of real data.

**Figure 6:**
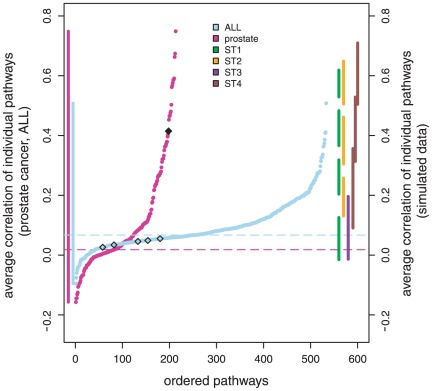
Average correlations for individual pathways for ALL (blue) and prostate cancer (violet) are shown by horizontally dashed lines. The two curves correspond to the rank ordered correlation values for ALL (blue) and prostate cancer (violet). For ST I (green - 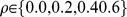), ST II (orange - 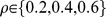), ST III (purple, 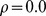) and ST IV (brown - 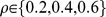) the projections of the range of correlation values is shown on the right-hand side.

A central topic of this paper is the investigation of the influence of the correlation strength and its structure on the identification of differentially expressed pathways. In the introduction we presented arguments supporting the need for such an analysis. Now we add quantitative evidence, directly extracted from the used expression data from prostate cancer and ALL. As discussed in the methods section ‘Surrogate data: ALL and prostate cancer’, both microarray data sets were normalized. Estimating the average correlation among all genes from the normalized data results in 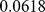 and 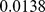 for ALL and prostate cancer, which are quite small correlation values. However, if we estimate the average correlation among all genes within *each pathway*, we obtain an entirely different result. In Fig. 6 we show these results by ordering the pathways according to their average correlation coefficient. The different number of the pathways results from the fact the we consider 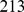 pathways for prostate cancer and 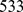 pathways for ALL, as explained in section ‘Surrogate data: ALL and prostate cancer’. Despite the fact that the average correlation among all genes is 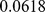 for ALL (blue) and 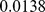 for prostate cancer (violet), shown as dashed lines in Fig. 6, one can clearly see that within the pathways there is a non-neglectable correlation, which spans a very wide range of different values, as summarized by the two vertical intervals on the left-hand side (violet: prostate cancer; blue: ALL). From these results, we can draw the following conclusions. First, even in normalized expression data there exist quite large correlations within particular pathways, which exhibit much larger values than the average correlation between all genes in the data set. The reason for this is that the purpose of any normalization method is to reduce reduce correlations due to technical artifacts and batch effects in the data but not real biological correlations between genes. These results justify also the selected correlation values for our simulations, which assume correlations up to 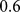. Second, there is a wide dynamic range of observed correlation coefficients that points toward a heterogeneity among the pathways. That means not all pathways possess the same characteristics but they can be quite different from each other.

For comparison with the simulated data, we include in Fig. 6 the correlation values for ST I to IV for different parameters. The vertical intervals on the right-hand side correspond to the projected correlation range of these four simulation types. Ordered from left to right: ST I (green: 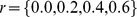), ST II (orange: 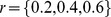), ST III (purple: 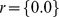) and ST IV (brown: 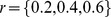). Here, we represent only the projected correlation range to simplify the presentation in Fig. 6. However, we would like to note that for each of these individual results the ordering of the correlation values assumes a similar shape as observed for prostate cancer and ALL. From these intervals, two observations are important to emphasize. First, ST I and II result in a shorter range for individual simulations, compared to ST III and IV. Second, the intervals for I and II are non-overlapping. Overall, we observe that if ST III and IV are employed together, the whole range of experimentally observed correlations found within normalized microarray data can be covered without gaps. Further, in comparison to the correlation structures used in previous studies, as discussed in the introduction, we find that ST III and IV provide a more realistic correlation structure compared to studies using a constant [13], [29], random [30], autoregressive [31], compound symmetry [14], [32], [33] or no correlations at all [25], [28].

We would like to point out that results about the range of the correlation values is only one indicator that should be met by simulated data. In addition, the structure among the genes is another important characteristics. Due to the fact that the data for ST III and ST IV are generated in a way that the inverse of the covariance matrix does reflect the independence relations that can be found in a protein network, this is another crucial difference to previous studies equipping our approach with a more realistic correlation structure.

**Figure 7:**
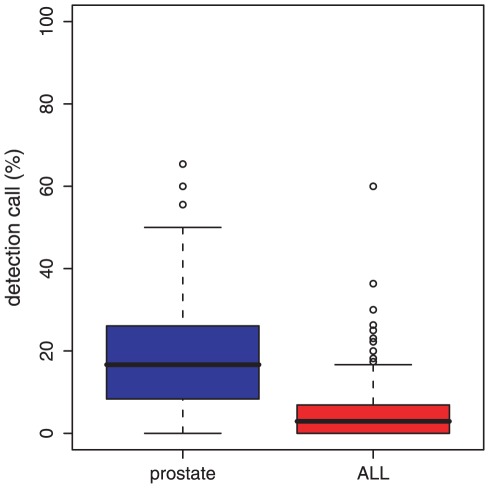
Distribution of the detection call (DC) values for gene expression data from prostate cancer (left) and ALL (right).

Finally, we present results about the biological distribution of DC values in prostate cancer and ALL. Using SAM [57] and a multiple hypotheses correction [58] we identify differentially expressed genes in prostate cancer and ALL for 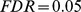. From this, we estimate a mean DC value of 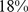 for prostate cancer and 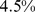 for ALL; see Fig. 7 for the distributions. Further, we find that only very few pathways have a DC value larger than 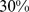, namely, 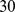 out of 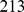 pathways (corresponding to 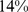) in prostate cancer and 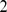 out of 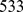 pathways (corresponding to 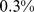) in ALL. This provides evidence that the selected DC values for our simulations correspond to biologically relevant values.

## Discussion

For our power analysis of simulated data, we assumed a maximum sample size of 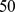 because this corresponds well to the number of samples available for the experimental microarray data we used. Further, most other microarray experiments conducted provide usually less than 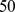 samples per condition making our choice from a biological point of view reasonable. Our results reveal the following. The *sum of t-square* test has for almost all studied cases the highest power if 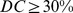, except for ST IV and 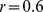. However, if 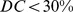 and 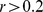 Hotelling's 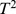 has a higher power. Due to the fact that this reflects the characteristics of the microarray data better, Hotelling's 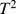 seems to be the favorable test. The *sum of t-square* test controls in general the FPR well, except for ST IV and 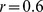. The control of the FPR of Hotelling's 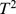 depends strongly on the pathway size, and a control is only working for pathway sizes less than 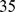. GSEA is for almost all studied cases underpowered, except for ST I and 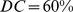 which is a condition that has not been found in one pathway in both microarray data sets. In our experience, this problem cannot be solved by increasing the sample size but is caused by *inappropriate* background data, which is out of the control of the experimenter. On the other hand, GSEA has a good control of the FPR for all conditions.

Taking all this into account our findings do not suggest to apply a method unconditionally to all pathways in a given data set, but to *filter* them in order to eliminate conditions for which a method is more likely to cause problems. We suggest to filter the pathways according to the following easy to check criteria: Hotelling's 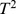 should only be applied to pathways with less than 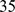 genes and a sample size larger than 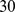. The *sum of t-square* test should only be used for pathways with 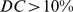 and a sample size of 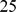 or larger. GSEA should only be used for pathways with 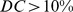 and a sample size larger than 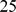. We want to emphasize that these sample sizes are different to the minimal sample sizes discussed in section ‘Surrogate data: ALL and prostate cancer’, which consider only the control of the FPR, whereas the optimal sample sizes avoid in addition that a study is underpowered. It is interesting to note that in [59] a similar sample size recommendation has been given, however, for the stability of clusters obtained from clustering algorithms. Despite the methodological differences among these studies, this correspondence is interesting because it emphasizes that there is a considerable difference between studies for detecting the differentially expression of (single) genes and studies for identifying differentially expressed pathways. For the former it would be plausible to expect lower sample size recommendations than for a clustering analysis trying to estimate the correlation strength among genes. However, due to the fact that our sample size recommendations for DEP methods *coincide* with clustering methods, hints, that DEP analysis methods are not *just* the sum of individual gene test statistics. If one perceives this problem from a biological perspective, this correspondence becomes more plausible because the clustering of genes is frequently used to reveal functional relations between genes corresponding to biological pathways [60]–[62]. Hence, clustering algorithms and pathway methods respond to similar molecular functional units.

The underlying rationale for our power analysis, which provides statistical estimates of the true positive rates of tests, is to study the unbiased performance of the methods. In contrast, by conducting multiple hypotheses tests one would be obligated to apply a multiple testing procedure to control a selected error measure [58], [63]. However, this would introduce a bias in the obtained results because both, the selected error measure and the control procedure, effect the results. In order to minimize this influence (it is probably not possible to completely eliminate this influence) one would need to study which pathway-based method works best together with which error measure and control procedure. However, these technical adjustments would not contribute to a better understanding of the power of a pathway-based method itself.

The optimal experimental design for microarray experiments with respect to the identification of the DEP is an important topic that is currently still under debate. From our comprehensive analysis of simulated and experimental gene expression data of over 3 million pathways, we obtain three major results. First, we find that the heterogeneity of different biological conditions and the sensitivity of the statistical methods suggest a selective application to definite pathways. That means, it is not advisable to apply a method to all accessible pathways but only to selected ones. Second, future gene expression experiments aiming to detect the DEP should be conducted with an increased number of samples in order to avoid non-robust and underpowered studies. From our study, we find method-specific recommendations constituting lower bounds for minimal sample sizes. Specifically, we suggest sample sizes between 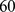 and 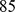 to avoid (1) an underpowered study and (2) to allow the control of the FPR. Third, as a more theoretical finding we gained insight into the correlation structure of biological and simulated microarray data. From these results, we suggest the combined usage of ST III and ST IV for simulating gene expression data. Because these simulation types lead to a more realistic correlation structure compared to studies employing a constant, a random or no correlation structure at all. On a side note, we would like to remark that by using simulation methods like *GeneNetWeaver* [64] or *SynTReN* [65], which are aiming to mimic the mechanistic behavior of the transcription regulation of genes, it is also possible to obtain simulated expression data with a realistic correlation structure. However, the generation of data from sampling is simpler and usually less time consuming. Further, the controlled, concerted modification of expression levels of genes in particular pathways may be very challenging for such methods.

For future studies of DEP methods, simulations based on our approach using ST III and ST IV can be very useful to investigate, e.g., the influence of different gene network structures, the effect of overlapping pathways or the influence of heterogeneous effect sizes. For example, one could compare protein networks and transcriptional regulatory networks for different organisms or compare them with gene regulatory networks. Here by gene regulatory networks we mean networks inferred from gene expression data [66]. Also different gene regulatory networks inferred from different inference methods [67]–[70] could be studied to investigate distinctions on the pathway level. Regarding overlapping pathways and their potential importance for pathway methods, such simulation settings provide ample opportunity to control parameters for testing hypotheses about their influence. Lastly, for our study we used a constant effect size for the differentially expression of genes. That means, we sampled differentially expressed genes for the control group from 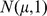 with 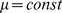. This is similar to all previous studies we are aware of, e.g., [13], [25]. However, it could be intricate to identify a distribution from which the mean 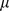 should be sampled.

The studied methods in this paper are expected to be also useful for the analysis of RNA-seq data [71], [72]. For this reason, once a sufficiently large data set is available, it would be interesting to repeat the above investigations for this new data type in order to gain a deeper insight into their experimental design in the context of DEP. Another important future direction to explore would be an investigation of the influence that alterations in regulation mechanisms in pathways have on the biological function [8].
